# Reimagining gendered community interventions: the case of family planning programs in rural Bangladesh

**DOI:** 10.1186/s41256-023-00337-8

**Published:** 2024-01-15

**Authors:** Bhanu Bhatia, Sarah Hossain, Upasona Ghosh, Fanny Salignac

**Affiliations:** 1 Faculty of Arts and Society, Charles Darwin University, 21, Kitchener Drive. Waterfront, Darwin City, Northern Territory 0800 Australia; 2 Freelance, Sydney, New South Wales Australia; 3 Indian Institute of Public Health, Bhubaneswar, West Bengal India; 4 TD School, University of Technology Sydney, Sydney, New South Wales Australia

**Keywords:** Social networks, Purdah, Gender, Family planning, Embeddedness

## Abstract

Family planning programs in Bangladesh have been successfully operating for over half a century, achieving phenomenal reductions in fertility rates. Acknowledging restrictions on women’s freedoms, much of the initial program design was concentrated on giving household supplies for women priority. However, one unfortunate impact of these outreach services is that, by bypassing the opportunity to challenge patriarchal attitudes directly, they inadvertently reinforce the power relationships of the status quo. Hence, we problematise the decision-making structures within Bangladesh’s family planning programs. We argue that the fundamental flaw with Bangladesh’s family planning program is the lack of conscious effort to understand women’s health choices and decision-making as a complex contextual process of relational, structural, and institutional forces. Additionally, avoiding men in these programs often creates new dependencies for women, as this approach does not directly seek to build relational bridges based on equality between genders. As a result, many women still depend on permission from their husbands and family for reproductive health services and face constrained family planning choices and access to care. We recommend that family planning programs adopt a broader vision to create new and more sustainable possibilities in an ever-evolving social relations landscape where gender is constantly negotiated. Such strategies are even more pressing in the post-Covid world, as national systems are exposed to uncertainty and ambiguity.

## Introduction

Through funding from global institutions such as the World Bank, community-based (CBD) programs have taken centre stage in delivering services, with family planning initiatives being a focal achievement of this approach [1–5]. The national family planning program of Bangladesh typified the most notable model of service delivery, one that directly targets women with services [1]. However, while this program has succeeded in reducing fertility rates, the evidence shows that it has not resulted in a radical change in gender norms—an outcome that was much hoped for [1, 3, 5–8].

Gender norms in Bangladesh are governed by patriarchy. A senior male heads the household, and descent and property are inherited through the male line, which means that women own nothing and are “genealogically irrelevant” [9]. Once married, women leave home to live with their husband’s family. Solidifying the patriarchal system further are the norms of *purdah*, which are adhered to by most women in Bangladesh. Purdah, which involves women wearing concealing clothing, effectively restricts women's mobility and keeps them economically dependent on men. Kabeer explains, “the overall consequences of these interacting constraints mean that not only is women’s access to material resources extremely limited, but their social interaction tends to be restricted to the ‘given’ relations of husband’s family” [9]. Entrenching the gender norms further is the notion of “connective selfhood” [10], “one that sees itself embedded in others and fosters relationality as a central charter of selfhood” [9]. This relational understanding of self often solidifies the norms of subordination for women as claims and obligations are articulated in the significant social relationships of kinship, family and community. The very same social relationships reinforce the dynamic that “power and privilege flow towards men” [9].

Ignoring the embeddedness of women in the relational web [9], priority is given to programs that deliver their services directly to women [3]. In tune with neo-liberal policies, reproductive health has become a favourite, with most initiatives adopting the direct delivery approach and promising to deliver beyond practical outcomes, such as reduced fertility and gender equality [2, 7, 9]. While fertility rates have dropped dramatically, reducing the burden of continuous childbearing on women, empowerment indicators provide mixed results [1, 7, 8]. The assumption was that lessening fertility due to contraceptive use would allow for a reduction in the amount of time and effort spent on raising kids by women. The availability of family planning is regarded as a prelude to greater freedom because it frees up time for the development of economic opportunities and wealth, elevating women's status within the family [8]. This would lead to more empowerment through improved employment opportunities, a greater presence in the public sphere, enhanced property rights, etc. Even though the fertility rate has decreased because of contraceptive use and decision-making in the household may have slightly improved, there has been little movement on other issues such as property rights^1^ [8, 11].

In most of these programs, female fieldworkers, through fixed and mobile community posts, target women in their homes [12]. The assumption is that such initiatives will empower women by providing practical resources and mobilising social support [9, 13]. However, a closer look into family planning programs exposes various pitfalls and only sporadic dedication to aims of empowerment, merely believing that population control will give women considerable freedoms, such as the freedom of movement in public places [6–8, 11]. Empowerment is a complex process, without a clear definition. Rooted in the concept of agency [14], empowerment is notoriously difficult to measure because it requires going from a disempowered to an empowered state along multiple dimensions. Nevertheless, in an empowerment process, women should feel entitled to make choices in the family, community, and society; negotiating autonomy in a complex web of relationships in both the public and the private domain [9]. Empowerment is, thus, a relational contextual construct that encapsulates the idea of choice and power. However, for many women in Bangladesh, the family, embedded in its patriarchal structure, is a central tenet of disempowerment.

Ignoring deep-seated questions of empowerment, aid has brough about a perspective on health that focusses on reaching the masses with support services. These movements are rooted in horizontal networks of support, with a cadre of professionals conveniently embedded in the communities in which they operate, reinforcing patron-client relations with a focus on services [4, 15]. Little in the program designs and implementation focus on women as individuals with rights to holistic reproductive health care and avenues to make full, accessible, and informed choices about effective birth control [6, 16]. The net result has often been a simple shift in dependencies—from one on the family group to one that now includes fieldworkers as advocates, information sources, and bridges to the outside world. Yet this model is called upon in the post-Covid world to reinvigorate the family planning agenda [17], even though COVID-19 exposed the inability of the patriarchal structures to negotiate any change that would lead to a dramatic drop in contraceptive rates [17, 18]. There is still much work to do to change the fundamental power relations in which women are embedded. Questions need to be raised about the sustainability of service-centric programs with limited capacity building and agility as part of their design [3, 11].

Clearly, the outreach model has not paid enough attention to the power relations women find themselves in, nor how these relationships continue to constrain women. Such programs have limits to engaging both sexes in imagining an equitable future. Further, a singular focus on service delivery adds another layer of dependency to an already complex relational structure that subjugates women; thereby, ignoring the vulnerabilities that challenge the long-term viability of such a program’s aims, especially in the face of a crisis. By bringing perspectives from diffusion studies on family planning and gender, we provide a new conceptualisation that provides pathways for reimagining the social landscape with an opportunity for new health policies to emerge for a sustainable future.

## Outreach and gender in family planning programs in Bangladesh

### The national family planning apparatus and dependency

The success of Bangladesh’s national family planning program has informed many contemporary CBD practices. Backed by international donors and NGOs, family planning services are ‘delivered to the door’ using thousands of full-time female fieldworkers, where women are visited in their households with a range of contraceptive services. Fieldworkers visit women based on age, reproductive stage, and contraception method, supported by satellite and community clinics for family planning services from a fixed site [19]. The prevalence of these services has meant that knowledge of contraceptives is now widespread in Bangladesh. The contraceptive prevalence rate (CPR) has also dramatically increased from 8% in 1975 to 62% in 2018 with a corresponding drop-in fertility rate from 6.3 to 2.3 children per woman. Further, most women know of at least one modern contraceptive method. However, the actual fertility rate exceeds the desired fertility rate of 1.7 [20]. The programs are also mainly women-centric; thereby, failing to provide Bangladeshi men access to services or counselling from the fieldworkers [20].

The Bangladeshi family planning program was conceptualised in the challenging socio-economic environment of extreme poverty and the poor status of women. Conventional wisdom at the time predicted that, in regions of extreme poverty and patriarchal control, improvements in women’s status to increase their decision-making capacity, especially in the area of sexuality and reproduction, was vital for fertility decline to occur [12]. Lacking these preconditions, door-to-door services were designed to: give women better access to contraceptives in their homes; create social support for fertility regulation; and to motivate couples to adopt family planning, all the while expanding women’s social networks and causing ideational change about the merits of a small family [12, 21]. Notably, female fieldworks and doorstep delivery has been credited as central to the drop in fertility rates [12]. However, a less than idyllic picture is revealed when we view the idea through its social–historical relationship structures. The patron-client model is still steeped in hierarchal kinship relationships—relationships legitimising access to differential resources [4]. For example, in the communities, the fieldworkers are called elder sisters. This incorporates existing hierarchical kinship structures into the relationships between patrons and clients. However, it also instils a power imbalance [15].

The fieldworkers themselves must reinterpret the meaning of purdah to reconcile their working conditions by appealing to concepts such as internal modesty rather than challenge the basic tenets of purdah. Even though health care is aligned with the symbolism of the gendered role of caring, the first fieldworkers were pioneers in entering paid employment outside the household and received substantial resistance. These field workers have been able to create a space for themselves in the public sphere by, first, providing quality care and resources to other women and, second, by adapting the meaning of purdah to one of modesty rather than seclusion. They also act as role models for other women, encouraging investments in education and opening the door for more women to enter the public sphere. That said, fieldworkers continue to play gendered roles at home. For example, they seek consent to leave home, use veils and coverings and have learned to carry on their household responsibilities and work outside, doubling their workloads [22].

In its prime in the 1990s, 40% of married women in rural areas reported a visit by a health worker sometime in the last six months [2]. This number has recently come down to 20% [20]. It is important to note that the visits are selective and do not include women who may not need contraception for reasons such as husbands living overseas. Policy changes towards fixed-site clinics have also resulted in reduced home visit. While community and satellite clinics are an important part of the outreach service, the most popular modern family planning method rural women use is the pill, which can be received at home [20]. The long-term socialisation of women in-home delivery has led women to the market for supplies with the aid of family. Approximately 10% of women used community and satellite clinics [20, 23, 24]. Since women have limited access to shops and markets, men provide a conduit between their wives and the pharmacy—not just for supplies but for instructions on taking the medication [23, 24]. Men also channel advice from health professionals to their wives [25].

Furthermore, even though the strategy places family planning front and centre of the agenda, it has failed to provide universal coverage, with 10% of women still unable to access contraceptive services [20]. Often without access to direct counselling, women face problems of ineffective use, high discontinuation rates, side effects, and method failure [20, 26]. A closer examination of reasons for discontinuation shows that while many women may have actively sought to discontinue due to desire for another child, issues in managing side effects and accidental pregnancies were important reasons for discontinuation, suggesting underlying struggles with the existing system in exercising full agency [20, 26]. Overall, 30% of pregnancies in 2011 were unintended [27]. Contraceptive failures and access issues have also led to unsafe abortions [5]. Several studies show that poor fertility outcomes are reduced if women have contact with fieldworkers [28].

These findings highlight the forgotten role of husbands in the supply chain and the immersion of household delivery in the psyche of rural life.

Existing challenges in the fragmented healthcare system in Bangladesh, such as: the availability of health workers and issues with managing and coordinating primary healthcare services were severely exacerbated during COVID-19 [18]. Family planning use decreased by 23% during the pandemic compared to the pre-pandemic level, and the use of oral contraceptives drastically dropped from 62 to 24% during the same period [18]. Several recent studies have recommended taking on sufficient family planning workers to restore full door-to-door services [17, 18]. However, the interplay of such policies with patriarchal structures fails to challenge the structures that inhibit women from exercising their reproductive rights in full.

### Outreach and empowerment

In a social setting where women’s domestic spheres are impenetrable, outreach workers provide key social capital, opening women to contact with the rest of the world [21]. It was assumed that giving women some control over their reproductive health would translate to increasing women’s negotiation power [7, 8, 11]. It was also presumed that lower fertility rates would free up women’s time and create opportunities for them outside the household [13]. However, understanding of the causal relationship between family planning and empowerment is still limited in the Bangladeshi context.

Evidence shows that the drop in Bangladeshi fertility rates has not coincided with the neo-classical assumption of greater workforce participation by women [13]. In fact, indicators of the labour force for fertile married women in areas of intense family planning outreach are significantly lower than for women in the other areas studied [7]. Peters [7] also finds that women living in areas with a relatively strong family planning program paid 14% more in dowries. More recent work confirms positive educational impacts and employment opportunities from the intergenerational impacts of family planning—that is, on the children of mothers practicing family planning—but altered migration patterns across the generations [29].

Drawing on data from the 1970s, Ruthbah et al. [8] show that lower fertility reduces decision-making power in the household and limits property rights.^2^ Yet, it increases mobility and access to economic resources. However, a study based on the data from 2006 shows women have gained in household decision-making, but freedom of mobility without permission remains contested. Only 13% of women report being able to attend a clinic or visit family or friends without seeking permission [11]—the primary impediment being travelling alone. More recent data indicates that mobility has improved over time, but the extent of this change is not entirely clear. For example, one indicator—whether women can visit a health clinic or hospital by themselves or with children—is included in the nationally representative Bangladesh Demographic and Health Survey (conducted in 2014) to assess women's freedom of mobility. While this variable shows that women’s mobility has increased, it is still challenging to evaluate because it is not obvious if women need to get permission to travel independently or not as per this variable. Furthermore, it is unclear how frequently women make use of the option to travel alone or with kids. This complexity becomes more apparent when we evaluate another indicator of decision making in the same survey, which shows only 14.1% of women reported making decisions regarding their own health care by themselves, and nearly 50% reported the decision was made jointly with their husband [30]. In a following national survey, nearly 70% of women stated that they made joint decisions about their health care, while only 9.7% of women claimed to be able to make the choice independently. Similarly, of the women currently using contraceptives, about 77% decided to do so with their husbands, and only 15.5% did so on their own [20]. Yet, the government of Bangladesh’s future vision is of more women travelling to community clinics for family planning, which ignores the impediments related to travel and exercising agency [11]. Instead, home visits and reliance on one’s husband for supplies have become central in reinforcing the system of purdah [20, 23]. The conclusion is that any improvement to the status of women resulting from outreach programs has come about because a couple wants to avoid an unwanted pregnancy rather than because of any ideational change in a woman’s position in society [31]. Not surprisingly, the literature shows mixed results when it comes to empowerment and family planning [7, 8, 11].

Because there is no mention of empowering women or giving them rights over their bodies, this door-to-door model of promoting contraception has found a harmonious existence in communities [5]. Further, in rural settings, the people feel a fundamental subservience to health workers [15]. This has further enhanced the strength of community clinics in creating an aura of power. For example, healthcare providers face limited resistance when insulting female clients and providing poor or limited services in clinics [5, 32, 33], although some of these behaviours may be due to heavy workloads.

It is important to emphasise that Bangladesh’s outreach services were conceptualised to control population growth; they were never conceptualised to challenge the discourse over women’s choices [5]. However, the United Nations’ human rights movement has recognised a critical lack of emphasis on women's reproductive health rights [16]. The movement cautions that we must move away from assumptions of women’s empowerment using birth control methods to one that directly tackle women’s ability to control their own reproductive health through ability to exercise autonomy, access to adequate services, information and infrastructure. Further, these processes need to be embedded in political, economic, social and cultural rights [16]. The reality of the Bangladeshi program is still a far cry from this vision [16].

We argue that its outreach intervention fails to penetrate the gendered decision-making structure surrounding Bangladesh's family planning. Fieldworkers providing contraceptives at the door may mean more women use family planning, but this does not challenge women to imagine a reality where they have the freedom and confidence to access the health services they are entitled to.

### Embeddedness

Recent research on embeddedness purports very similar conclusions—women’s choices are still constrained in rural Bangladesh, and they are still highly dependent on men but now also include fieldworkers.

Embeddedness is operationalised using the concept of social networks, which views social relations, such as friendship, as a dominant factor in an actor’s decision-making. Several studies show that large-scale diffusion of birth control practices mainly occurs through interactions between family, friends, and neighbours [21, 34]. Women embedded in dense interconnected networks are found to be receptive to peer pressures and are only likely to use contraceptives if they have approval from other members [35]. On the other hand, women with more diffuse networks are less likely to fall subject to social pressure and are likely to use their networks to tap into social information and other required resources.

Bangladeshi women have been found to be receptive to family planning, openly sharing information and supplies with each other [33]. Yet their networks are dominated by social processes and have a gendered structure. A systematic mapping of network ties shows that, while rural women in Bangladesh are embedded in dense structures in the domicile, men form ties that are loosely structured well beyond the household with limited scope for normative pressures [21, 35]. Men have been found to network across household boundaries, which is conducive to interactions between diverse individuals. They also seem to dodge social pressure and are more effective in cross-gender interactions [21, 35]. Not surprisingly, however, women prefer to seek the approval of other women before adopting a family planning regimen [35].

Moreover, these women’s networks are steeped in structural issues of power. The findings from a quantitative study in the Matlab region of Bangladesh [21], which has been subject to a long-standing and intensive family planning program, show that women are not only embedded in unequal fragmented support networks but that the fieldworkers dominate the entire structure as they provide the only source of connectivity between women in different households. Thus, without a strong health program, women’s networks provide limited opportunities for information flow between different parts of the network. So, while it has been argued that an important indirect byproduct of these networks of workers is an ideational change in the role of women [35], the results only confirm the “stickiness” of cultural practices.

A much older study by Kincaid [36] deliberately challenges the domicile structure of women’s networks in Bangladesh. The study shows that women participating in family planning discussions beyond the household are more likely to use contraception. In another study, socially-oriented organisations have been found to be key in forming and transforming women’s relationships with each other from passive ties to “communities of practice”. These communities of practice then engage in challenging gender norms and offer support by invoking an ethos of solidarity, friendship, reflective practices, learning, and awareness [9]. Bangladesh has also opened itself to world markets, creating more opportunities for women to participate in the public sphere. However, such issues have been sidelined when it comes to policymaking. The family planning policy has meant that women continue to negotiate in dense isolated pockets of ties, with the only bridge to information being health workers. Meanwhile, men continue to seek guidance from among their own much more expansive networks—networks that are not necessarily well-informed about family planning. Worse still, if the fieldworkers are taken away, a woman’s only recourse to information from interpersonal sources outside the home is through their husband [21]. In a climate of purdah, this gives men a central role in the connected whole.

Even if today's world moves around technology and telecommunication, the digital divide between the genders is still prominent. According to a recent study done by Raihan, Uddin and Ahmmed [37], women and girls from rural Bangladesh are much less likely to own a mobile phone than their male counterparts. The discrimination widens further if the woman is older and has low literacy. Moreover, women are only indirectly connected to information-rich ‘bridging networks’ through their ‘bonding networks’ to a male [38]. Thus, digital network building is also dominated by gender roles and norms.

Growing evidence shows that men too can ascribe to equitable norms [39] and take increased responsibility for contraception [40]; however, they have not been included in the conversation. We also know that men themselves seek knowledge about contraceptives from their own networks and that many wish to take greater control of their own fertility choices [40–42]. The calls to include men are not new. Men require access, information and counselling regarding sexual and reproductive health [42]. These services can be integrated into the current service delivery model while connected to the social space men inhabit [41–43].

In Africa, where community-based family planning programs have been more inclusive of both sexes, health workers have been able to penetrate men’s networks and successfully influenced men’s preferences toward birth control [44, 45]. Findings show that when Bangladeshi men have a high peer interaction regarding family planning, a couple’s odds of using contraceptives increases by 2.7 [43]. Also, the integration of men into the current infrastructure in Bangladesh can be facilitated through methods such as staff training, raising awareness, group discussions and service provision [42].

Understanding the relational dimensions of decision-making can create pathways for challenging the dominance of men and, in particular, the postures that perpetuate gender inequality. In Bangladesh’s social hierarchy, men are superior to women, but that does not mean they have no choice over whether they oppress women. It may be a very limited choice, given the lack of exposure to alternative information, but it is still a choice, and this opens a door for change [39]. We argue that family planning programs should widen their exposure to men to promote more equal decision-making in family planning.

A recent program in Western Africa follows a novel approach in that key influencers are identified at the community level. The goal is to break down some of the barriers that prevent men and women from accessing family planning services. Creating pathways for change, the program’s principles are built around: identifying and mobilising influential actors in the communities; building gender-inclusive spaces for reflective dialogue that help to overcome barriers in communication; and balancing the program’s aims with the principles of gender equity [45]. This intervention is rooted in the network principles of ideational change through key influencers. Similar trials in neighbouring India targeted male gender ideologies in reproductive health programs without any adverse impacts and witnessed a strong retention rate in the program [46]. In rare cases when men have been explicitly targeted in Bangladesh interventions, it has improved understanding of women’s health issues [47]. The success of the Bangladeshi program in its patriarchal context is testimonial to community support [22]. Plus, there is direct evidence that men desire smaller families and that there is substantial potential for men to play a supportive role in family planning programs in Bangladesh [20, 35].

Most men and women do not have a fixed position on family planning issues. Monumental gains have been noted in reports of contraception discussions amongst Bangladesh couples and spousal agreement on fertility preferences [20]. Bargaining within a marriage is common, and couples often navigate key decisions by negotiating with each other [48]. Contraceptive use is no exception [40, 49]. It is, therefore, not surprising that communication and the power dynamics within a marriage have been found to be instrumental in dispelling misconceptions and changing attitudes about family planning in several contexts [40, 44]. Hence, a more nuanced approach to exploring ideas about fertility in a socially interactive world with multi-layered relationship structures is required.

Social relations are core to FP decisions, empowerment, and one’s ideas of self and body. Acknowledging these enmeshed realities allows for new dimensions to emerge that simultaneously link gender and family planning. For instance, efforts to transform women’s relationships with each other and men to share equal responsibility are hardly a threat to family planning efforts.

## Pathways of change

Bangladeshi family planning has achieved remarkable success in making the use of contraceptives a norm. Also, formidable resilience has been shown by women facing impediments to use family planning—increasing their burden to avail a basic right [50]. We, thereby, argue that the fundamental flaw with Bangladesh’s family planning program is the lack of conscious effort made to understand women’s health choices and decision-making as a complex contextual process of relational, structural, and institutional forces. Family planning programs are embedded in the social structure of society. The current ‘women only’ approach only serves to deepen the divide between the genders instead of exploiting the available pathways for change. This is not to say that there have not been credible gains in reducing fertility rates, but this alone is not enough to challenge deep-seated inequalities.

Interventions by fieldworkers to gain social acceptance for family planning methods have, for a long time, attempted to change the decision to use modern contraceptives from one made within a relationship to one of an autonomous decision. However, through these successful interventions by health workers to lower fertility rates, women have emerged as users of contraceptives rather than gaining the power of relational autonomy. The door-to-door service of health workers may alter conditions for rural women superficially, but this approach lacks the grounding to improve the position of women in the family and community. Eventually, keeping women confined to the home means that even the welfare service providers will not be successful in promoting personal autonomy, as their access to avail themselves of that service properly is conditioned on the choice of others’, not their own.

**Fig. 1:**
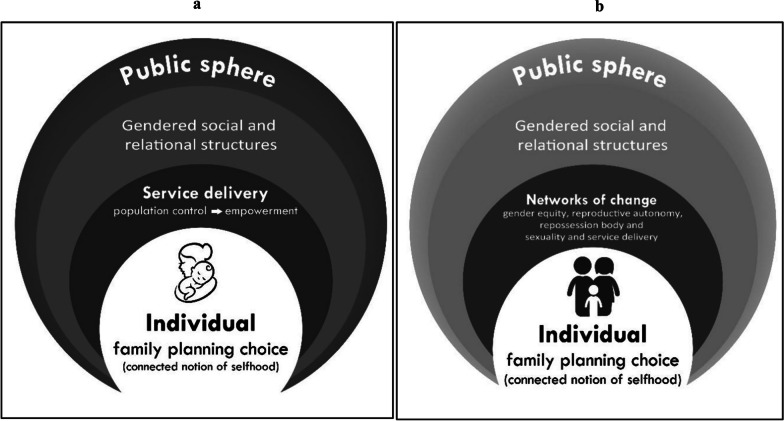
Family planning in the social relations landscape. **A** Contemporary model of family planning. **B** Proposed model of family planning service provision

Figure 1a shows the constrained set of choices available to women. As shown, the service delivery is embedded in a larger social structure that is shaped by an unequal gendered space and further surrounded by the boundaries of the public sphere, which itself is gendered [9].

The most prominent achievement of the family planning program is that women’s welfare has improved through access to contraception with their husband’s approval. Assumptions of gains in empowerment through population control are only partially supported by evidence. Nowhere in the program has there been any discussion of women reclaiming their bodies and challenging gender norms to exercise choices in shaping their well-being. Including women in family planning programmes has tended to focus on objective targets without any emphasis on the meaning of participation or the aim of reducing gender inequality. In a climate of limited opposition by men to family planning, the program fails to capitalise on opportunities for reflection by men on the power relations at play in a couple’s reproductive health responsibilities.

As Kabeer [9] points out, “acknowledging the value and significance of social relationships in people’s lives is very different from an uncritical perspective of the ‘station and duties in life’ ascribed to them”. Men and women may gain their sense of identity and personhood through family and kinship while growing up, but it is not until they experience an alternative that they can truly evaluate their relationships [9]. One can choose a community that reinforces one’s ingrained forms of inequality. Or choose to expand their knowledge and interact with a wider group, which may “open up the possibility of alternative ways of living that were hitherto inconceivable” [9]. Thus, no matter how deeply embedded women and men may be in the relationship structures, a critical assessment of one’s ties is still possible, providing space for the self to engage in new realities and reshape the social fabric [9].

Viewing family planning programs as embedded in an ever-changing canvas of social relations, where gender is negotiated in a contested space, creates multiple possibilities for new paradigms to emerge. Programs seeking to imagine a different reality need to move beyond ‘women only’ or ‘men only’ creating synergies for an equitable and sustainable future. Directly augmenting women’s status in terms of their immediate relations is likely to have a more self-sustaining impact on fertility than an indirect path through simple service provision [51]. Including men and building women’s confidence and creating pathways for expanding their ties beyond the home, thereby, giving women more movement in the public space to access services and greater social support emerge as important aspects of transformative structures.

The moment for this is rife with family norms changing and Covid-19 playing havoc with field visits. Cultural shifts in family planning as a norm, better transport systems, increasing acceptance of women in the public sphere, and mobile phone coverage all provide opportunities to realign current community-based approaches to the principles of gender equity [20, 22, 38].

With the realisation that individuals exist within social structures and empowerment is inextricably linked to family planning choices, we propose active engagement of men and women, with these two aspects as a starting point. Figure 1b illustrates a reimagined family planning service that engages with a relational reality, along with the principles of gender equity, to build supportive networks of change that can provide a critical perspective to men and women. This proposed model acknowledges that individual men and women are influenced at multiple levels. Furthermore, the provision of family planning services for both men and women are embedded in groups of change that permeate the boundaries of gendered structures and renegotiate the meaning of being male and female.

Powered by donor funds, armies of outreach workers seek to make rural Bangladeshi women independent by bringing financial, health, and social services to their doorstep. The model of service delivery has received widespread acclaim despite the issues of gender imbalance that are embedded and reinforced in social relationships. While new pathways are identified in this research, future research is needed to apply the proposed model practically that operationalises policy, services, community, and individual interventions. Lastly, more work is needed to capture the link between empowerment and family planning that considers contemporary conditions.

## Data Availability

This research did not involve any primary research or data analysis.

## Footnotes

1 The term property rights are used broadly to capture women's ownership of assets and inheritance rights.

2 The term property rights are used broadly to capture women's ownership of assets and inheritance rights.
